# Simplified Bayesian method: application in cytogenetic biological dosimetry of mixed n + γ radiation fields

**DOI:** 10.1007/s00411-018-0764-3

**Published:** 2018-11-21

**Authors:** I. Słonecka, K. Łukasik, K. W. Fornalski

**Affiliations:** 1 Central Laboratory for Radiological Protection, Konwaliowa 7, 03-194 Warszawa, Poland; 2 Faculty of Physics, Warsaw University of Technology, Koszykowa 75, 00-662 Warszawa, Poland; 3 Ex-Polon Laboratory, ul. Podleśna 81a, 05-552 Łazy, Poland

**Keywords:** Bayesian analysis, Cytogenetic biological dosimetry, Mixed radiation dosimetry, Retrospective dosimetry, Health physics, Radiation biophysics

## Abstract

This article describes the application of a simplified Bayesian method for estimation of doses from a mixed field using cytogenetic biological dosimetry, taking as an example neutron and gamma radiation emitted from the MARIA nuclear research reactor in Poland. The Bayesian approach is a good alternative to the commonly used iterative method, which allows separate dose estimation. In the present paper, a computer program, which uses the iterative and simplified Bayesian methods to calculate mixed radiation doses, is introduced.

## Introduction

Cytogenetic biological dosimetry includes a group of methods based on assessing the frequency of biomarkers, such as dicentric chromosomes in human peripheral blood lymphocytes, to estimate radiation doses of those who have been exposed in occupational accidents or incidents (IAEA 2011). Dose estimation with the dicentric assay after accidental exposure to mixed, i.e., neutron and gamma radiation (n + γ) is more complex than estimation after exposure to a non-mixed field, because the human body is irradiated by a combination of two (in general *R*) radiation types. Due to a markedly different relative biological effectiveness (RBE) of components of the mixed absorbed dose, there is a strong need to estimate not only the total absorbed dose, but also its components. In the case of mixed neutron and gamma radiation, if the ratio *ρ* of neutron (*D*_n_) to gamma (*D*_γ_) absorbed doses:

1 $$\rho =\frac{{{D_{\text{n}}}}}{{{D_{{\varvec{\upgamma}}}}}}$$

is known from physical measurements, the absorbed doses of each component can be obtained by applying an iterative process and assuming that all radiation-induced dicentrics are the sum of dicentrics induced by each radiation type (IAEA 2011). The iterative method can be generalized and presented in one mathematical formula as the “analytical method” (Słonecka et al. 2018). The estimation of the separate absorbed doses is performed using in vitro dose–response calibration curves for each type of mixed radiation. In the case where a physical estimate of *ρ* is not precisely known, the above method is not possible to use. However, Brame and Groer derived a method based on Bayesian statistics (Brame and Groer 2003), which allows estimating the neutron and gamma absorbed doses with an uncertain *ρ* with the use of prior distribution(s), known as the probability distribution function (PDF), which can be assigned to the unknown parameter. Therefore, the Bayesian method is an attractive alternative to the currently used classical methods. However, the Bayesian method is more complicated in comparison with the iterative one and requires use of advanced mathematical calculations. Because sometimes the only unknown parameter is *ρ*, and because calibrated curves are usually known from earlier measurements, the authors propose simplification of the original Brame and Groer method. More specifically it is proposed to assign the prior distribution only to the *ρ* parameter, and to express curve parameters as constant values instead of distributions. It is shown here that this approach greatly simplifies the calculations and gives comparable results.

The objective of this paper is to present the mentioned simplified Bayesian approach for dose estimation using the results of dicentric assay analysis after accidental overexposure to mixed n + γ radiation, which was implemented into a computer program, and to compare the results with those obtained by the full Bayesian method proposed by Brame and Groer (2003) and by the classical (iterative) method (IAEA 2011, Słonecka et al. 2018). Besides the mixed doses calculation, the developed program gives also the possibility of fitting curves to the data with the use of the robust Bayesian fitting (Fornalski et al. 2010; Fornalski 2015; Fornalski and Dobrzyński 2015), which has been used to find the dose–effect calibration curves and its parameters.

## Materials and methods

### Mixed n + γ radiation field

The equations of the neutron and gamma dose–response models can be written as (IAEA 2011):

2 $${Y_\gamma }({D_\gamma })={Y_0}+\beta ~{D_\gamma }~+\gamma ~{D_\gamma }^{2},$$

3 $${Y_{\text{n}}}({D_{\text{n}}})={Y_0}+\alpha ~{D_{\text{n}}},$$

where *Y*_x_ (where x = γ for gamma and x = n for neutron radiation) is the frequency of dicentrics, *D*_x_ is the absorbed dose, ${Y_0}$ is the control dicentric level (background), *α* and *β* are the coefficients of the linear terms while *γ* is the coefficient of the quadratic term. Assuming additivity of neutrons and gamma rays in dicentrics production (IAEA 2011, Kellerer and Rossi 1978), the dose–response relationship for mixed n + γ radiation may be described as:

4 $${Y_{{\text{n}}+\gamma }}({D_{\text{n}}},{D_\gamma })={Y_0}+\alpha ~{D_{\text{n}}}~+~\beta ~{D_\gamma }~+\gamma ~{D_\gamma }^{2}~ \equiv ~{y_{\text{f}}},$$

where *y*_f_ = *u*/*w* is the frequency of dicentrics, and *u* is the number of dicentrics observed in *w* analyzed cells (lymphocytes). It is assumed that the number of dicentrics per cell observed in a sample after neutron and gamma exposure follows a Poisson distribution with a population mean, *y*_*f*_ (IAEA 2011). In order to calculate absorbed doses it is also necessary to know the neutron to gamma absorbed dose ratio *ρ* (Eq. 1). Instead of $\rho$, the *θ* parameter can also be used,

5 $$\theta =\frac{{{D_\gamma }}}{{{D_\gamma }+{D_{\text{n}}}}}=\frac{1}{{\rho +1}},$$

because it proved to be much more convenient in calculations (Brame and Groer 2003). The *θ* is normalized to the [0, 1] range, whereas $\rho$ lies between 0 and ∞.

Having values of *Y*_0_, *α, β* and *γ*, the number of dicentrics observed in an analyzed lymphocytes sample and the *θ* parameter, the absorbed doses can be calculated using the *Y*(*D*) function (Eq. 4). If the *θ* is known from measurements, the classical iterative (IAEA 2011) or analytical method (Słonecka et al. 2018) can be used. In other cases, the Bayesian (Brame and Groer 2003), quasi-Bayesian (Słonecka et al. 2018), or even the Monte Carlo approach (Powojska et al. 2018) can be applied. The iterative method is described in detail in (IAEA 2011). It involves performing several series of calculations using the same input data. Each next series gives more accurate results, until the results in the following steps begin to repeat. This method is recommended by the International Atomic Energy Agency (IAEA 2011).

### Bayesian approach to dose calculation

#### General approach

Bayesian statistics express the final posterior function based on both, the prior function (*p*) and the likelihood function (*L*). All of them are expressed in the form of the probability distribution function (PDF). Thus, the prior PDF is utilized to estimate the most probable values of unknown parameters (here *θ* or *ρ*—the ratio of absorbed doses). Such a prior function [here: *p*(*θ*) or *p*(*ρ*)] is widely used in Bayesian statistics. Data obtained in the experiment are expressed formally as a likelihood function. In biological dose assessment, *L* can be found based on biophysical arguments, as a distribution of damages, which is given by Poisson statistics (IAEA 2011) and can be written as:

6 $$L=\frac{{{{(w~{y_{\text{f}}})}^u}~{e^{ - w~{y_{\text{f}}}}}}}{{u!}}.$$

Consideration of these two sources (*p* and *L*) is the foundation of Bayes’ statistics and allows transforming the prior PDF into a posterior PDF according to the Eq. 7:

7 $${\text{Posterior}} \propto {\text{Likelihood}}~ \times {\text{Prior}}=L \cdot p.$$

#### Full Bayesian method—the Brame and Groer approach

In the original Brame and Groer approach, proper prior functions are used for the *ρ* or *θ* parameter as well as for the *α, β* and *γ* calibration curves parameters. Brame and Groer assumed that prior PDFs of curves parameters can be approximated by the Gamma distribution (the logarithm of a Gamma distribution has a simple form for analysis, i.e., for the maximum likelihood method):

8 $$p(\lambda )={\lambda ^{k - 1}}\frac{{{z^k}}}{{\Gamma (k)}}{\text{exp}}~( - z\lambda ),~~$$

where *λ* = {*α, β, γ*} are the dose–response curves parameters, and *Γ* is a Gamma function. Parameters *k* and *z* are the shape and scale parameters, respectively, different for different *λ*. With such an assumption, the posterior function of dose can be written as:

9 $$P\left( {{D_{\text{x}}}} \right) \propto \mathop \smallint \nolimits^{} \mathop \smallint \nolimits^{} \mathop \smallint \nolimits^{} \mathop \smallint \nolimits^{} L\left( {{D_{\text{x}}}{\text{|}}\alpha ,\beta ,\gamma ,\theta } \right)~p\left( \alpha \right)~p\left( \beta \right)~p\left( \gamma \right)~p\left( \theta \right)~{\text{d}}\alpha ~{\text{d}}\beta ~{\text{d}}\gamma ~{\text{d}}\theta.$$

**Fig. 1:**
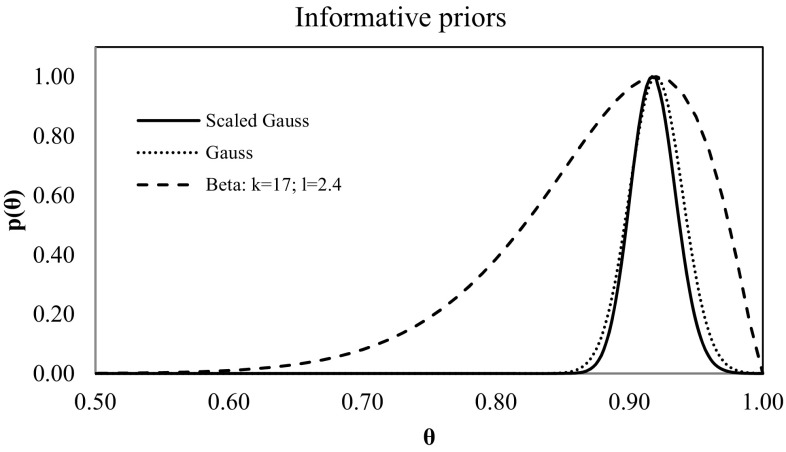
The informative priors for θ=0.92

The likelihood function, *L*, is given by the Poisson distribution (Eq. 6). Parameters *λ* = {*α, β, γ*} are given in the classical way with some standard deviation. Priors *p*(*λ*) can be introduced by a Gamma distribution each, like in Eq. 8, or obtained in another way, for example, by the robust Bayesian regression analysis (Fornalski and Dobrzyński 2015) (which would enhance the Brame and Groer method and make it Bayesian in all aspects). The *θ* parameter (described by Eq. 5) is given in the transformed form of a scaled Gaussian distribution (Fig. 1):

10 $$p(\theta )=\frac{1}{{\sqrt {2\pi } {\sigma _\rho }{\theta ^2}}}{\text{exp}}~\left[ {\frac{{ - 1}}{{2\sigma _{\rho }^{2}}}{{\left( {\left( {\frac{1}{\theta } - 1} \right) - \hat {\rho }} \right)}^2}} \right],~$$

where $\hat {\rho }$ is the expected value, ${\sigma _\rho }$ the standard deviation, and *θ* a variable of distribution.

One has to note, however, that Brame and Groer have used the Gamma distribution for priors *p*(*λ*) quite arbitrarily, to simplify their calculations. Their method, as mentioned above, can be enhanced and all priors *p*(*λ*) can be presented as distributions obtained by the robust Bayesian regression method (Fornalski 2014, 2015). This enhancement would change the Brame and Groer method into a Bayesian one in absolutely all aspects (also in the origin of priors), but would then require advanced numerical methods.

#### Simplified Bayesian method

The methodology presented above, but with several modifications (Pacyniak et al. 2014), has been successfully implemented at the Central Laboratory for Radiological Protection (CLOR), Poland. Assuming that in a practical situation the only unknown parameter is *ρ* (or *θ*), while calibrated curves are known from earlier measurements, the difference in both methodologies pertains to the expression of calibration curves parameters (*α* ± *Δα, β* ± *Δβ, γ* ± *Δγ* and *Y*_*0*_ ± *ΔY*_*0*_*)*. In the simplified Bayesian method, these parameters are expressed as fixed values instead of distributions, so the method does not use their uncertainties directly in dose calculations, but only in the assessment of dose uncertainty. Taking parameters as fixed values, the prior PDF can be assigned to the *ρ* parameter only, which greatly simplifies the mathematical calculation of dose and still gives comparable results. Additionally, the faster and simpler method is of crucial importance when a real nuclear accident happens and there is no time for more complicated calculations. More than that, most cytogenetic laboratories have their own calibration curves and the exact values of parameters are pretty well-known, so it is not necessary to transform them into probability distributions to use the full Bayesian method.

Therefore, in the method proposed here, transforming Eq. 7 into the form convenient for calculations, the probability distribution of absorbed dose can be expressed as:

11 $$P\left( {{D_{\text{x}}}} \right)=\mathop \smallint \limits_{0}^{1} L\left( {{D_{\text{x}}}|\theta } \right)~p\left( \theta \right){\text{d}}\theta,$$

where x = {γ, n}. Potential candidates for prior PDFs, *p*(*θ*), are detailed below. The likelihood functions for both absorbed doses, presented in the form of Eqs. 12 and 13, were obtained by the substitution of Eqs. 4 and 5 into Eq. 6:

12 $$L(\theta )=\frac{{{{[w({Y_0}+\alpha \frac{{1 - \theta }}{\theta }{D_\gamma }+\beta {D_\gamma }+\gamma D_{\gamma }^{2})]}^u}}}{{u!}}~ \cdot {e^{ - w\left( {{Y_0}+\alpha \frac{{1 - \theta }}{\theta }{D_\gamma }+\beta {D_\gamma }+\gamma D_{\gamma }^{2}} \right)}},$$

13 $$L(\theta )=\frac{{{{\left[ {w\left( {{Y_0}+\alpha {D_{\text{n}}}+\beta \frac{\theta }{{1 - \theta }}{D_{\text{n}}}+\gamma {{\left( {\frac{\theta }{{1 - \theta }}{D_{\text{n}}}} \right)}^2}} \right)} \right]}^u}}}{{u!}}~ \cdot {e^{ - w\left( {{Y_0}+\alpha {D_{\text{n}}}+\beta \frac{\theta }{{1 - \theta }}{D_{\text{n}}}+\gamma {{\left( {\frac{\theta }{{1 - \theta }}{D_{\text{n}}}} \right)}^2}} \right)}}.$$

The curve parameters can be assessed in advance by maximum likelihood estimation or even by the robust Bayesian regression method (Fornalski 2015). Below the differences between the results of those methods are presented.

The uncertainties of dose estimations, *σ*_*D*x_, can be assessed using the Cramér–Rao theorem:

14 $${\sigma _{{D_{\text{x}}}}} \geqslant \frac{1}{{\sqrt {|\frac{{{\partial ^2}{\text{ln}}~(P(Dx))~}}{{\partial D_{{\text{x}}}^{2}}}|} }},$$

where ln(*P*) is the natural logarithm of *P*(*D*_x_) due to the maximal likelihood method. However, the variable presented in Eq. 14 is the lower bound^1^ of the variance of the estimator, so it can underestimate the uncertainty. Thus, the classical assumption, like the independent finite increments method, can be applied as well.

In practice, all presented calculations need numerical solutions, because analytical solutions are too complicated in some cases.

#### Informative prior functions

The prior function for *ρ* (or *θ*) used in the Bayesian method should reflect the actual knowledge about the ratio of absorbed doses. Prior PDFs may be presented in the informative or non-informative form. To select the proper prior, information about the *θ* (or *ρ*) parameter, such as the expected value of the parameter, needs to be considered. Based on the detailed information available, the prior function (with its scale and shape parameters, in some cases) should be taken to maximize its PDF for the considered *θ* (*ρ*) parameter. Information about the parameter can be derived, for example, from the standard operation of a nuclear reactor (level of nuclear fuel burn-up for instance), that is a main source of an intense mixed n + γ radiation field.

The authors have tested different PDFs, both informative and non-informative priors. The most practical exemplary priors are presented below, in form of Eqs. 15–20.

The Gaussian distribution (Eq. 15) is usually selected in biodosimetry worldwide because the estimation of *θ* (or *ρ*) is prepared using the classical Gaussian regression method around $\hat {\theta }$:

15 $$p(\theta )=\frac{1}{{\sqrt {2\pi } {\sigma _\theta }}}{\text{exp}}[ - \frac{{{{(\theta - \hat {\theta })}^2}}}{{2\sigma _{\theta }^{2}}}]~,$$

where *σ*_*θ*_ is the standard deviation, $\hat {\theta }$ the expected value, and *θ* the variable of the distribution (Fig. 1).

The Gaussian prior together with Eq. 5 gives the scaled Gaussian prior (Eq. 10), where $\rho =\frac{1}{\theta } - 1$. As it has been mentioned earlier, this PDF has been proposed and tested by Brame and Groer, because it fits better to the *θ* parameter than to *ρ*.

Likewise, the Beta distribution (Eq. 16) with the appropriate shape parameters can be also used as an informative prior (Fig. 1):

16 $$p(\theta )=\frac{{\Gamma (k+1)}}{{\Gamma (k)\Gamma (l)}}{\theta ^{k - 1}}{(1 - \theta )^{l - 1}}~,$$

where *Γ* is the gamma function, $k$ and $l$ are distribution shape parameters, and *θ* is the variable of distribution.

While the Gaussian (or scaled Gaussian) prior seems to be obvious as a standard for biodosimetry, due to its symmetrical form (Słonecka et al. 2018), the unsymmetrical form of the Beta distribution can be a good solution for criticality accidents, where the stream of neutrons can be potentially higher than expected.

#### Non-informative prior functions

In special cases, even a non-informative prior PDF can be used, although such a prior does not specify the exact information, it only defines a very general trend of the parameter. Such a prior can be used in the rare case of the lack of detailed information about the dose ratio. A non-informative prior can also be used in situations when a significant contribution of one type of radiation is assumed, but the exact value of *ρ* is not known (Słonecka et al. 2018). For example, having the information that during the standard operation of a nuclear reactor there is an overwhelming dominance of gamma ray over neutrons, the non-informative distributions can be useful.

In some cases, the only available information is that there was mixed radiation composed of two (generally *R*) radiation types, e.g., a neutron and gamma radiation field, with an unknown *θ*. In such situations, one can try to assess the absorbed doses assuming approximately a similar contribution from neutrons and gamma rays, which corresponds to the PDF expressed as the simplified Beta distribution (Eq. 16) converted into Eq. 17 (for the parameters $k$ = *l* = 2):

17 $$p(\theta ) \propto (\theta - {\theta ^2}).$$

**Fig. 2:**
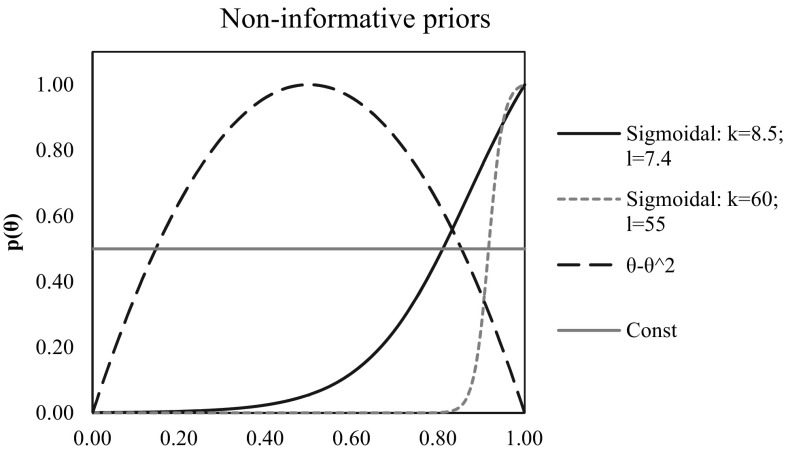
The non-informative priors

Such a form of the Beta distribution (Fig. 2) can potentially describe a situation with an uncontrolled fission reaction, i.e., a criticality accident in a fuel pool, where the neutron fluence can be high.

Equation 18, hereinafter referred to as the sigmoidal prior function, can be used as a non-informative prior function (Fig. 2). The sigmoidal form of a prior PDF can represent the situation of an uncontrolled fission reaction in a fuel pool or the shutdown of a reactor, where usually gamma rays are dominating, but some neutrons may also be present.

18 $$p(\theta )=\frac{2}{{1+{\text{exp}}( - k\theta +l)}},$$

where $k$ and *l* are shape parameters, and *θ* is the variable of the distribution.

The simplest non-informative prior is the constant one (Eq. 19) (Fig. 2):

19 $$p(\theta )={\text{const}}.$$

This prior represents the situation with completely no information about the irradiation conditions (which is rather an academic scenario). The use of such a prior gives the least precise results whenever used. This is the reason why it is important to always try assessing the potential shape of prior, which is usually possible. Selection of the correct prior PDF depends on the situation. In the present paper, several distributions are proposed and used in the calculating program, but there might be additional ones.

#### Proper prior selection

Selection of the appropriate prior function is of crucial importance in Bayesian methods. This function reflects the existing knowledge from other sources/assumptions, which is the main difference between the classical and Bayesian statistics approach. Therefore, all additional information available shall be connected with the proper prior function.

The Gaussian (or scaled Gaussian) prior function given by Eq. 15 (or Eq. 10, respectively) is commonly used in cytogenetic biodosimetry (Brame and Groer 2003; Ainsbury et al. 2013a, b) due to its symmetry. This symmetry is reflected in the simple normal distribution of data around $\hat {\theta }$. However, in some cases, this symmetry is not perfectly appropriate. For example, during some scenarios of nuclear accidents, one can assume a lack of knowledge about one type of radiation, e.g., neutrons. In that scenario, there is completely no information about the emission of neutrons, but their existence cannot be excluded. Therefore, a sigmoidal prior shape would be a better option because it describes that situation properly: no neutrons at all or some emission of neutrons are on equal footing.

It is emphasized, however, that the selection of an accurate prior function depends on the user of the algorithm. It is thus subjective, because the user has the best knowledge about any additional information on the radiation scenario to be investigated. In most cases, the Gaussian function might be first choice as it is the simplest one.

### Generalized multi-field of radiation

The method presented above works well if two radiation fields are present. If more (*R*) fields of different types are present, however, it is necessary to find a more general solution. The required multi-field Bayesian approach was originally formulated by Fornalski (2014).

For the general case of many types of radiation, Eq. 4 can be presented as (Fornalski 2014):

20 $$Y({D_{{\text{total}}}})={Y_0}+\mathop \sum \limits_{{i=1}}^{R} \mathop \sum \limits_{{j=1}}^{\mu } {\lambda _{i,j}}D_{i}^{j},$$

where *µ* is the degree of the *i*-th polynomial and *R* is the number of radiation types present. In practice, however, usually *µ* is less than or equal to 3, which is be assumed in the following considerations. Additionally, one can assume that each *i*-th polynomial has the same degree. Thus, for such the generalization of the simplified Bayesian method for dose estimation it is necessary to assume (*R*-1) parameters *θ*_*i*_ for each radiation type *i* (Eq. 21) and proper prior functions, *p*(*θ*_*i*_).

21 $${\theta _i}=\frac{{{D_i}}}{{\mathop \sum \nolimits_{{\xi =1}}^{R} {D_\xi }}}=\frac{{{D_i}}}{{{D_{{\text{total}}}}}}.$$

A prior density function should be assumed or established experimentally, as in Eqs. 15–19. While making a choice, for the benefit of simplicity, the polynomial Eq. 20 can present *y*_*f*_ dedicated for the exact *i*-th dose as (Fornalski 2014):

22 $${y_{\text{f}}}({D_i}) \equiv Y({D_i})={Y_0}+\mathop \sum \limits_{{i=1}}^{R} \mathop \sum \limits_{{j=1}}^{\mu } {\lambda _{i,j}}{\left[ {\frac{{{\theta _i}}}{{1 - {\theta _i}}}\left( {\left( {\mathop \sum \limits_{1}^{R} {D_\xi }} \right) - {D_i}} \right)} \right]^j}.$$

Each absorbed dose *D*_*i*_ is written as a proper part of the total dose using Eq. 21 and the reasoning used in Eqs. 12 and 13. Next, the likelihood function based on the Poisson distribution (Eq. 6) and *y*_*f*_ (*D*_*i*_) from Eq. 22 can be written as (Fornalski 2014):

23 $$L({\lambda _0},{\lambda _1}, \ldots ,{\lambda _{\mu R}},{\theta _1},{\theta _2}, \ldots ,{\theta _{R - 1}})~ \propto ~~{[{y_{\text{f}}}({D_i})]^u}~ \times ~{e^{ - ~w~~{y_{\text{f}}}({D_i})}}.$$

Assuming that all fitted parameters, *λ*, are given by their priors (including *λ*_0_ = *Y*_0_), the posterior probability distribution for each absorbed dose equals (Fornalski 2014):

24 $$P\left( {{D_i}} \right) \propto \mathop \smallint \nolimits^{} \ldots \mathop \smallint \nolimits^{} ~L\left( {{D_i}{\text{|}}{\lambda _{0, \ldots ,\mu R}},{\theta _{1, \ldots ,R - 1}}} \right) \cdot \left( {\mathop \prod \limits_{{j=0}}^{{\mu R}} p\left( {{\lambda _j}} \right){\text{d}}{\lambda _j}} \right) \cdot \left( {\mathop \prod \limits_{{\xi =1}}^{{R - 1}} p\left( {{\theta _\xi }} \right){\text{d}}{\theta _\xi }} \right),$$

which is the most general form of posterior probability for *R* types of radiation and *(µ+1)R* numbers of fitted parameters.

### Computational program

A number of authors have suggested Bayesian methods as an appropriate tool in biological dosimetry (Brame and Groer 2003; Ainsbury et al. 2013a, b; Pacyniak et al. 2014) and some of them have written and shared programs, like *CytoBayesJ* (Ainsbury et al. 2013b), utilizing the Bayesian approach. This is a very elaborate and user-friendly program which (1) enables testing for the most appropriate model for the distribution of chromosome aberrations amongst cells; (2) allows calculating the posterior probability distribution for the yields of chromosome aberrations; (3) allows calculating the probability distribution of radiation dose using a “Bayesian like” method with linear and linear-quadratic dose response models; (4) offers full Bayesian calculations of the probability of radiation dose for Poisson data using a linear dose response model; and (5) offers Bayesian methods to calculate decision thresholds and detection limits (Ainsbury et al. 2013b). All of the above functions require very complex and complicated mathematical operations, so the software is also very complex.

In contrast, in the present paper it was the intention to develop a simple tool that is easy to use. Consequently, the program proposed in the present paper allows for calculation of separate doses of the components of a mixed radiation field composed of low- and high-LET radiation (here n + γ) using a simplified Bayesian approach. The software contains a list of prior PDFs which can be used. In addition, the program offers a few additional options including (1) calculating mixed doses using classical (iterative) and analytical methods; (2) fitting parameters to the data with the use of robust Bayesian method (Fornalski 2014, 2015; Fornalski and Dobrzyński 2015); and (3) automatically choosing the better among two possible dose response curves: linear or linear quadratic (Fornalski and Dobrzyński 2015).

In general, despite allowing for many applications, the program is very easy and intuitive to use. The software includes a graphic user interface and is freely available^2^. The program does not require knowledge of complicated mathematical expressions, which are of course needed and used by the program behind the scenes to perform the dose calculations and their uncertainty. The program was written in the Java programming language. Its advantage is the *EXE file extension, which gives the possibility to install it on almost every computer. The beta version of the program is being continually developed. In case of any issues or questions, please contact the authors.

## Results and discussion

To test the program, results of a dicentric assay analysis performed in CLOR were used (Pacyniak and Kowalska 2015). In the CLOR experiment, human blood samples were irradiated in vitro in the H8 channel of the MARIA nuclear research reactor in the National Center for Nuclear Research (Otwock-Świerk, Poland) in a mixed neutron and gamma-ray field. From physical measurements, it was known that the field consisted mainly of gamma rays and thermalized fission neutrons. To determine the total dose components, the twin detector method and recombination method were used (Golnik et al. 2013). The dose fraction recommended as the contribution of gamma rays to total tissue kerma was 0.92 ± 0.02. Hence, the dose fraction of thermal neutrons to the total tissue kerma was 0.08 ± 0.02. Thus, the *ρ* = *D*_n_/*D*_*γ*_ = 0.087.

### Fitting curves parameters

The parameters of the calibration curve were calculated with the use of maximum likelihood estimation (MLE) and the robust Bayesian method (BM) (Fornalski 2014, 2015; Fornalski and Dobrzyński 2015), and they are compared in Table 1. As one can see, both methods give similar results with small differences in uncertainties. Generally, the Bayesian method works better in the case of outlier points (Fornalski 2015; Fornalski and Dobrzyński 2015), because it simply omits such points and finds the best curve fit to scattered/biased data. More detailed information about this method is given in (Fornalski 2014, 2015; Fornalski and Dobrzyński 2015).

**Table 1:** Fitted parameters for the dose response curves (Eqs. 2–4) calculated with the Bayesian method (BM) and maximum likelihood estimation (MLE). *Y*_0_—background level of dicentrics; α and β—parameters of linear term of dose response; γ—parameter of quadratic term of dose response

Source		Method	*Y*_0_ ± σ_*Y*0_^*^ [dic ⋅ cell^−1^]	α, β ± σ_α,β_^*^ [dic ⋅ cell^−1^⋅Gy^−1^]	γ ± σ_γ_^*^ [dic ⋅ cell^− 1^⋅Gy^− 2^]
Mixed radiation field, n + γ		BM	0.0010 ± 0.0001	0.038 ± 0.001	0.048 ± 0.002
		MLE	0.0010 ± 0.0001	0.038 ± 0.004	0.048 ± 0.003
Neutrons		BM	0.0005 ± 0.0001	0.354 ± 0.002	–
		MLE	0.0005 ± 0.0001	0.354 ± 0.003	–
Gamma radiation: ^60^Co		BM	0.0010 ± 0.0001	0.011 ± 0.001	0.056 ± 0.001
		MLE	0.0010 ± 0.0040	0.012 ± 0.003	0.056 ± 0.002

^*^Uncertainties are presented as one standard deviation

### Mixed doses assessment—comparison of full and simplified Bayesian methods

To compare the full Bayesian method and the simplified Bayesian method developed here, the data tested by Brame and Groer (2003) were used. These data are related to an accident scenario simulated for the French nuclear reactor SILENE (Voisin et al. 1997). In that scenario, the blood was irradiated by reference pulses of mixed gamma and neutron radiation. The used data are characterized by the following parameters: *u* = 85 dicentrics, *w* = 28 cells, *ρ* = 0.64 ± 0.25, *α* = 0.835 ± 0.098 dic⋅cell^− 1^⋅Gy^− 1^, *β* = 0.0142 ± 0.0098 dic⋅cell^− 1^⋅Gy^− 1^, *γ* = 0.0759 ± 0.0126 dic⋅cell^− 1^⋅Gy^− 2^, and *Y*_0_ = 0. In the simplified Bayesian method developed in the present paper, the scaled Gaussian prior (Eq. 10) for the *θ* parameter was used (where *θ* = 0.61 ± 0.25) as well as the fixed values of the curve parameters and the assumption about the Poisson distribution of aberrations in analyzed cells. In contrast, in the full Bayesian method (Brame and Groer 2003), the scaled Gaussian prior for *θ* was used and the *α, β* and *γ* parameters were expressed in the form of Gamma distributions. Both methods gave consistent results and were compared to the iterative method (Table 2) recommended by the International Atomic Energy Agency (IAEA 2011).

**Table 2:** Comparison of results obtained with the iterative, full Bayesian and simplified Bayesian methods

Method	*D*_γ_ [Gy]	^*^RE [%]	*D*_n_ [Gy]	^*^RE [%]
Iterative	3.670 ± 1.975	–	2.349 ± 1.342	–
Simplified Bayesian^**^	3.617 ± 1.134	1	2.287 ± 0.733	3
Full Bayesian^***^	3.635	1	2.318	1

^*^RE relative error, the difference between iterative and each next dose

^**^Scaled Gaussian prior was used to calculate the doses

^***^Results were presented in (Brame and Groer 2003) in form of graphs without uncertainties

### Mixed dose assessment—simplified Bayesian method

To estimate neutron and gamma absorbed doses separately, a sample with 35 dicentrics in 500 analyzed cells was used, the fitted curve parameters were taken from Table 1, and *θ* = 0.92 ± 0.02. The results of the iterative and simplified Bayesian methods are shown in Table 3, as well as the absorbed doses, which were measured during the experiment. In the simplified Bayesian method only a few priors, as discussed above, were used.

**Table 3:** Results of mixed dose assessment. In the case of the simplified Bayesian method several priors, both informative (INF) and non-informative (NON-INF) have been used

Method		*D*_γ_ [Gy]	^*^RE [%]	*D*_n_ [Gy]	*RE [%]
Physical		0.782 ± 0.040	–	0.068 ± 0.003	–
Iterative		0.796 ± 0.093	2	0.069 ± 0.008	1
Simplified Bayesian^**^ PRIORS					
INF	Gauss *θ* = 0.92	0.798 ± 0.103	2	0.071 ± 0.016	4
	Scaled Gauss *θ* = 0.92	0.798 ± 0.096	2	0.071 ± 0.014	3
	Beta *θ* = 0.92	0.760 ± 0.184	3	0.112 ± 0.041	65
NON-INF	Sigmoidal *θ* > *0.8*	0.900 ± 0.129	15	0.060 ± 0.025	12
	Sigmoidal *θ* > *0.5*	0.840 ± 0.225	7	0.146 ± 0.043	115
	*θ–θ* ^2^	0.199 ± 0.199	75	0.182 ± 0.034	168
	Constant	0.225 ± 0.354	71	0.185 ± 0.035	172

^*^RE relative error, the difference between actual–physical value of dose and each next dose

^**^See Figs. 1and 2 for more details

In the situation where the *ρ* (or *θ*) parameter is known from physical measurements, both the iterative and simplified Bayesian methods give comparable results (Gaussian, scaled Gaussian or Beta priors). Typically, the Bayesian method is used when a physical estimate of the composition of the mixed field was not made (or when its composition is not exactly known). Then, more detailed information would be useful, such as the dominance of one radiation type over another. In such a case choosing of a proper prior PDF, even non-informative, would be important. As it is shown in Table 3, even non-informative prior functions, (especially the sigmoidal priors here) can give consistent results. In case of the Beta distribution (*θ–θ*^*2*^) and a constant prior, where only general information is available, the resulting doses are different as compared to the physical absorbed doses (Table 3) and, consequently, they are not proper priors in this situation. They were used to show that any information about the event that occurred may be very important. The constant prior, which represents a situation where information about the irradiation conditions is completely lacking, gives the least precise results. This is the reason why it is important to always try assessing the potential shape of the prior PDF, which is usually possible.

## Conclusions

In this study, a simplified Bayesian method was developed for biodosimetry applications in mixed radiation fields, as an alternative to the full Bayesian approach described in (Brame and Groer 2003) and to the iterative approach described in (IAEA 2011, Słonecka et al. 2018). The proposed method assumes the use of fixed values for the parameters of dose–response curves instead of their distributions, which can be applied in situations when the parameters are known from earlier physical measurements, which is a very common situation in practice. As a major advantage, the approach proposed here makes the method faster and simpler, and much more adequate to real experimental/clinical situations, which is, for example, of crucial importance during real accident scenarios.

The results of all methods investigated here are comparable (depending on the used prior function). Despite the implemented simplifications, the method developed here is still complicated. Therefore, a code with the Bayesian approach was implemented in the form of a computational program, which does not require knowledge of complex mathematical operations. The code is freely available, and the software is presented in a simple form with a graphical interface. It allows calculating mixed absorbed doses in fields composed of low- and high-LET radiation. Furthermore, the code contains a number of priors, which were tested in the present study. Depending on the situation, some of these priors can be successfully used, especially the informative ones, like Gaussian, scaled Gaussian and Beta distributions. Some of the non-informative priors, like sigmoidals, also provided reasonable results. Although the neutron absorbed doses obtained with the sigmoidal prior PDFs differ from the physical ones (like the prior for *θ* > 0.5), gamma doses were assessed with good precision. Even this is a very useful information despite the prior PDFs used being imprecise. Having knowledge about the total absorbed dose, which can be calculated by the iterative method (IAEA 2011) with the use of the mixed curve parameters (Table 1) and the gamma absorbed dose, the neutron absorbed dose can be easily calculated. As it was mentioned above, the least precise information was given by the constant prior PDF.

The program developed has even more options, as it also enables doses calculation using the iterative (IAEA 2011) and analytical methods (Słonecka et al. 2018). It also allows fitting linear or linear quadratic curves to data and automatically identifies the best-fitting model using the Bayesian model selection algorithm (Fornalski and Dobrzyński 2015).

Future studies will focus mostly on multi-field irradiation with more than two radiation types. Such a situation can happen in some rare nuclear accident scenarios, terrorist attacks or during space travel. The last case seems to be the most important because of future plans of Mars exploration, where radiation protection aspects will be of crucial importance (Greco et al. 2003; Testard and Sabatier 1999).

## Footnotes

1 The Cramér–Rao theorem gives lower bounds of the variances of the estimators, that are just the elements of the diagonal of the inverse of the Fisher information matrix. These lower bounds are asymptotically attained by the variances of maximum likelihood estimators, and the inverse of the Fisher information matrix can be estimated from the inverse of the Hessian matrix, *H*^− *1*^, of the log-likelihood function evaluated at the maximum likelihood estimates.

2 http://www.clor.waw.pl/publikacje.html.
